# Mu-opioid receptor and delta-opioid receptor differentially regulate microglial inflammatory response to control proopiomelanocortin neuronal apoptosis in the hypothalamus: effects of neonatal alcohol

**DOI:** 10.1186/s12974-017-0844-3

**Published:** 2017-04-14

**Authors:** Pallavi Shrivastava, Miguel A. Cabrera, Lucy G. Chastain, Nadka I. Boyadjieva, Shaima Jabbar, Tina Franklin, Dipak K. Sarkar

**Affiliations:** The Endocrine Program, Department of Animal Sciences, Rutgers, The State University of New Jersey, 67 Poultry Lane, New Brunswick, NJ 08901 USA

**Keywords:** MOR, DOR, Microglia, Inflammatory and anti-inflammatory cytokines, Fetal alcohol, POMC

## Abstract

**Background:**

Opioid receptors are known to control neurotransmission of various peptidergic neurons, but their potential role in regulation of microglia and neuronal cell communications is unknown. We investigated the role of mu-opioid receptors (MOR) and delta-opioid receptors (DOR) on microglia in the regulation of apoptosis in proopiomelanocortin (POMC) neurons induced by neonatal ethanol in the hypothalamus.

**Methods:**

Neonatal rat pups were fed a milk formula containing ethanol or control diets between postnatal days 2–6. Some of the alcohol-fed rats additionally received pretreatment of a microglia activation blocker minocycline. Two hours after the last feeding, some of the pups were sacrificed and processed for histochemical detection of microglial cell functions or confocal microscopy for detection of cellular physical interaction or used for gene and protein expression analysis. The rest of the pups were dissected for microglia separation by differential gradient centrifugation and characterization by measuring production of various activation markers and cytokines. In addition, primary cultures of microglial cells were prepared using hypothalamic tissues of neonatal rats and used for determination of cytokine production/secretion and apoptotic activity of neurons.

**Results:**

In the hypothalamus, neonatal alcohol feeding elevated cytokine receptor levels, increased the number of microglial cells with amoeboid-type circularity, enhanced POMC and microglial cell physical interaction, and decreased POMC cell numbers. Minocycline reversed these cellular effects of alcohol. Alcohol feeding also increased levels of microglia MOR protein and pro-inflammatory signaling molecules in the hypothalamus, and MOR receptor antagonist naltrexone prevented these effects of alcohol. In primary cultures of hypothalamic microglia, both MOR agonist [D-Ala 2, N-MePhe 4, Gly-ol]-enkephalin (DAMGO) and ethanol increased microglial cellular levels and secretion of pro-inflammatory cell signaling proteins. However, a DOR agonist [D-Pen2,5]enkephalin (DPDPE) increased microglial secretion of anti-inflammatory cytokines and suppressed ethanol’s ability to increase microglial production of inflammatory signaling proteins and secretion of pro-inflammatory cytokines. In addition, MOR-activated inflammation promoted while DOR-suppressed inflammation inhibited the apoptotic effect of ethanol on POMC neurons.

**Conclusions:**

These results suggest that ethanol’s neurotoxic action on POMC neurons results from MOR-activated neuroinflammatory signaling. Additionally, these results identify a protective effect of a DOR agonist against the pro-inflammatory and neurotoxic action of ethanol.

**Electronic supplementary material:**

The online version of this article (doi:10.1186/s12974-017-0844-3) contains supplementary material, which is available to authorized users.

## Background

Microglia, macrophage-like cells of the brain, are a type of innate immune cell in the central nervous system (CNS). Under normal physiological conditions, microglia are known to secrete neurotrophins and protective cytokines to promote neuronal development and survival. However, upon CNS insult or injury, microglia can acquire complex phenotypes in order to participate in the cytotoxic response, immune regulation, and injury resolution. The classical M1-type activation is associated with cytotoxicity and inflammatory responses, while the alternative M2 activation is regarded as being beneficial [1]. It has been shown that chronic alcohol drinking activates microglia to an M1 phenotype and promotes inflammatory response [2]. Activation of microglia to the M1 phenotype is particularly detrimental during the developmental period, as this may lead to neurotoxicity and developmental disorders [3].

Studies in adult animal models of alcohol abuse provide clues to the effects of ethanol on microglia [4]. In these models, ethanol administration induces an activated morphological phenotype and stimulates production of pro-inflammatory and neurotoxic molecules. In an adolescent animal model, alcohol exposure increases the risk for persistent and long-lasting increases in brain neuroimmune gene expression and neurodegeneration [5]. Studies in fetal and neonatal animal model show that ethanol may stimulate neuron cell death, at least in part, through stimulation of neuroinflammatory and neurodegenerative processes in the CNS [6]. In primary cultures, chronic alcohol increases the release of pro-inflammatory cytokines and oxidative stress molecules from microglia and reduces intracellular cAMP and brain-derived neurotrophic factor in co-cultures of hypothalamic neurons and microglia. Alcohol-activated microglia-conditioned media increases apoptosis in immature hypothalamic neurons [7–9]. Neonatal alcohol exposure in rodents induces neurotoxicity in hypothalamic neurons in vivo [10]. Alcohol exposure affects the viability of neurons following neonatal alcohol exposure, and peroxisome proliferator-activated receptor-γ (PPAR-γ) agonists limit this ethanol-induced cell loss [11]. Repeated alcohol exposure during the developmental period may also lead to long-term sensitization of microglia that result in persistent pro-inflammatory signaling in the brain following insult [12].

Alcohol exposure has many detrimental effects on the developing brain and has been known to cause fetal alcohol spectrum disorders (FASD). Many FASD patients show lifelong stress response abnormalities, as demonstrated by augmented responses to stress hormones such as adrenocorticotropin and corticosterone [13, 14]. Studies have shown that the abnormalities observed in the stress response of prenatally ethanol-exposed rats appear to be driven by alterations in the functions of the hypothalamic-pituitary-adrenal (HPA) axis [15], partly due to reduction in the number and function of stress regulatory beta-endorphin (BEP) producing proopiomelanocortin (POMC) neurons in the hypothalamus [10]. The neurodegenerative effect on POMC neurons by developmental alcohol is connected with alcohol activation of microglia and resultant neuroinflammation [8, 12].

Recently, it has been shown that microglial activity is tightly controlled by communication between neurons and microglia both under healthy and pathological conditions. Under stress, neurons may release immunomodulatory factors and signaling molecules including neurotransmitters, which may recruit microglia proximally to the affected neurons and induce activation of microglia [16–19]. Mechanisms for the bi-directional communication between activated microglia and neurons may include numerous types of neurotransmitter receptors which are present on microglia [20]. Because BEP, an opioid peptide, acts via mu- (MOR) and delta- (DOR) opioid receptors, the possibility is raised that these receptors are involved in the communication between POMC neurons and microglia during ethanol toxicity.

Using in vivo neonatal alcohol feeding rat model and in vitro primary cultures of rat hypothalamic microglia and neural stem cell-derived POMC cell models, we evaluated the role of MOR and DOR in ethanol activation of microglia to promote apoptotic action on POMC neurons. We provide evidence that alcohol neurotoxic action on POMC neurons results from differential expression and action of MOR and DOR to promote MOR-activated neuroinflammatory signaling and to reduce DOR-regulated anti-inflammatory signaling in microglia.

## Methods

### Animals

Adult Sprague-Dawley rats were obtained from Charles River Laboratories (Wilmington, MA). Adult transgenic mice (C57BL/6J) expressing the fluorescent protein EGFP in POMC neurons were obtained from Dr. Malcolm Low’s laboratory at Oregon Health & Sciences University, Portland. All animals were kept under standard lighting conditions (12-h lights on; 12-h lights off) and provided rodent chow and water ad libitum. These rats and mice were bred to generate neonatal animals, which were used in this study. The postnatal day 1 old rat pups were used as the source of fetal rat hypothalamic tissue to prepare microglial cultures. In vivo studies were conducted by feeding rat or mice pups with oral gavage of a milk formula containing 11.34% ethanol (vol/vol) twice daily at 2 h intervals, yielding a total daily ethanol dose of 2.5 g/kg (AF), fed with isocaloric control (PF), or they were left in the litter with the mother (AD) as described by us previously [10]. The feedings were conducted at 10:00 AM and 12:00 PM daily for 5 days (Postnatal days 2–6). This dose of ethanol gives rise to blood alcohol concentrations of approximately between 150–200 mg/dL. After each feeding, the pups were immediately returned to the litter. Some of the AF animals were additionally received daily s.c treatment with minocycline (45 μg/kg; 1 H prior to the first feeding), MOR antagonist naltrexone (NTX, 10 mg/kg; 15 min prior to the first feeding), or DOR agonist naltrindole (NTD, 10 mg/kg; 15 min prior to the first feeding). All these drugs were purchased from Sigma-Aldrich (St. Louis, MO). Two hours after the last feeding on postnatal day (PND) 6, some of the pups were sacrificed and the mediobasal hypothalamus (the mediobasal portion of the hypothalamus extended approximately 1 mm rostral to the optic chiasma and just caudal to the mammillary bodies, laterally to the hypothalamic sulci, and dorsally to 2 mm deep) was dissected and used for microglia extraction by optiprep gradient separation method or frozen for measurement of protein or gene measurement. Other pups were transcardially perfused, their brains collected, postfixed, cryoprotected, frozen, and cut into 30 μm coronal sections for immunocytochemical studies.

### Primary microglial culture

Microglial cells were prepared from PND 1 rat pup hypothalamus (both sexes) using the method described by us previously [8]. Cells were plated at 2 × 10^5^ cells/cm^2^. Cultures were fed every 4 days with DMEM/MEM/Hams F12 (HDMEM) in a 4:5:1 ratio with 10% fetal calf serum (FCS). On day 12, the culture was shaken on a rotary shaker at 800 rpm for 1 h. The suspended cells were plated on uncoated T25 flasks and incubated for 1 h at 37 °C. Then, the medium containing suspended cells was discarded and adherent cells were fed with HDMEM for 3 days to develop the microglial culture. To confirm the purity of isolated microglial cells, the culture was stained with IBA-1, a microglial marker or the astrocyte marker glial fibrillary acidic protein (GFAP), and visualized under microscope. The isolated microglial cultures were 99% IBA-1-positive cells considered as pure microglial culture. Microglial cells were maintained in DMEM-F12 with 5% FBS in 24-well plates (1 × 10^5^ cells/well) until experimentation. Prior to treatment, microglial cells were fed with DMEM-F12 containing serum supplement (DMEM-F12, 30 nM selenium, 20 nM progesterone, 1 μM iron-free human transferrin, 100 μM putrescine, and 5 μg/ml insulin).

Microglial cells were treated with various doses of ethanol (25–100 mM), [D-Ala 2, N-MePhe 4, Gly-ol]-enkephalin (DAMGO; 50 μM) with or without ethanol (50 mM), [D-Pen2,5]enkephalin (DPDPE; 10 nM) with or without ethanol (50 mM), naltrexone (10 ng/ml) with or without ethanol (50 mM), naltrindole (50 μM) with or without ethanol (50 mM), DAMGO (50 μM) with ethanol (50 mM) and naltrexone (10 ng/ml), DPDPE (10 nM) with ethanol (50 mM) and naltrindole (50 μM), or vehicle for 24 h. All chemicals were purchased form Sigma-Aldrich (St. Louis, MO). After 24 h, media from treated microglial cells were collected, centrifuged, and used for POMC neuronal apoptosis studies or stored at −80 °C for multiplex ELISA and cells were harvested for extraction of proteins. In the immunoneutralization study, microglial cells were pretreated with various neutralizing antibodies (1 ng/ml of anti-TNF-α, T3198, Sigma-Aldrich, St. Louis, MO; 0.5 ng/ml of anti-IL-6, AF506, R&D System, Minneapolis, MN; 5 ng/ml of anti-IL-13, MAS-23735 or 1 ng/ml of anti-IL-4, BVD4-1D11 both from Thermo Fischer, Rockford, IL) 1 h prior to the treatment with ethanol with or without MOR and DOR agonists. After 24 h of incubation, media from treated microglial cells were collected, centrifuged, and used for POMC neuronal apoptosis study.

### Preparation of POMC cells from neural stem cells

Enriched POMC neuronal population were prepared by differentiation of neural stem cells in vitro by the methods described by us previously [21]. In brief, pregnant rats of the Sprague-Dawley strain at 18 to 20 days of gestation were sacrificed, and the fetuses were removed by aseptic surgical procedure. The brains from the fetuses were immediately removed; the hypothalami were separated and placed in ice-cold Hanks’ balanced salt solution containing anti-biotic solution (100 U/ml penicillin, 100 μg/ml streptomycin, and 250 ng/ml amphotericin B), 0.1% bovine serum albumin, and 200 μM ascorbic acid (all from Sigma-Aldrich, St. Louis, MO). The hypothalamic cells were washed and then incubated at 37 °C for 5 min using the same medium. After dispersion, the cells were plated at a density of 3.0 × 10^6^ cells per 25-mm^2^ flask and at a density of 1.0 × 10^6^ cells per well in a 24-well plate. Both the flask and plate were coated with polyornithine at a concentration of 100 μg/ml and then incubated for 3 h. The cells were maintained in Dulbecco’s modified Eagle’s medium with 10% fetal calf serum at 37 °C and 7.5% CO2 in a humidified water-jacketed incubator for 2 days. On day 2, the medium was replaced with HDME containing 10% fetal calf serum, 33.6 μg/ml uridine, and 13.2/ml 5-fluorodeoxyuridine (Sigma-Aldrich, St. Louis, MO) to stop the overgrowth of glial cells. Then, cells were used for the isolation of stem cells for a period of 3 weeks (see for more details, [21]). These neurospheres were differentiated by treating these cells for 1 week with PACAP (10 μM; SynPep) and dibutyryl cAMP (cAMP; 10 μM; Sigma) in serum-free, chemically defined medium (HDME consisting of 30 nM selenium, 20 nM progesterone, 1 μM iron-free human transferrin, 100 μM putrescine, and 5 μg/ml insulin) and then maintaining them in defined cell culture medium without the drugs for 1 week. These differentiated cells were all stained for POMC derived peptide BEP. During experimentation, POMC cells were cultured (10×^6^ cells/well) in T25 flasks for 2 days. The cells were then exposed to conditioned medium from microglia activated with ethanol with or without opioid agonists and antagonists or vehicle for a period of 24 h. Following this cells were lysed with nucleosome lysis buffer and run for nucleosome assay using nucleosome ELISA kit (Calbiochem, USA) for determination of POMC neuronal apoptosis.

### In vivo microglial separation and flow cytometry analysis of proteins in microglia

Microglial cells were isolated from the mediobasal hypothalamus of PND 6 pups (both sexes) from three neonatal pups using Optiprep density gradient and methods described previously [22] with some minor modifications. Briefly, mediobasal hypothalamic tissue samples were isolated and mechanically dissociated using 18-gauge needle followed by a 21-gauge needle in Hank’s balanced salt solution (HBSS) media (Sigma). The cells were strained with 40 μm cell strainer and trypsinized (0.5% trypsin) to digest tissues. The trypsinization reaction was stopped by adding HBSS + 10% FBS media. The cells were strained to get rid of myelin and then loaded on an optiprep column. Optiprep columns were prepared by diluting optiprep with MOPS (3-(N morpholino) propanesulfonic acid) buffer (0.15 M NaCl, 10 mM MOPS, pH 7.4). The diluted optiprep is again diluted in different proportions as 35, 25, 20, and 15% in HBSS media. These solutions were then loaded in a series as most dense on bottom and least dense on top 35, 25, 20, and 15%. Isolated cells were then loaded on top and the columns were centrifuged at 1900 rpm for 15 min at 20 °C. The microglia and red blood cells (RBC) gathered into a pellet at the bottom of the column. The pellets were taken and incubated with 0.85% ammonium chloride to lyse RBCs. The remaining purified microglia were washed with 1× PBS 2 times and fixed with 4% paraformaldehyde for 10 min. The cells were then stained for IBA-1 (microglial marker), GFAP (astrocyte marker), and MAP2 (neuronal marker) to determine the purity of microglia. The isolated microglia were >90% pure.

These purified microglia were used for protein measurements. This was done by flow cytometry analysis. For this, isolated microglia were stained with primary antibodies, rabbit anti-IBA-1 (1:100; Wako Pure Chemical Industries, USA), rabbit anti-DOR (1:100; Santa Cruz, Billerica, MA), rabbit anti-MOR (1:100; Antibodies Inc, Atlanta, GA), mouse anti-TLR4 (1:100; Abcam, Cambridge, MA), rabbit anti-P-38 MAPK, IκBα, P-JNK, and P-AKT (1:100: Cell signaling, Danvers, MA), and mouse anti-NF-κB (1:100, Millipore, Billerica, MA). The cells were labeled with FITC-488 secondary antibody (1:400, Abcam, Cambridge, MA) respective to their primary host and then analyzed by BD Accuri C6 Flow Cytometry. Five thousand events per sample were read for all samples, and data analysis was completed with C6 Accuri software. Flow cytometric gates were set using unstained cells using the forward scatter and side scatter plot, and labeled cells were read on the FL-1A (488) channel. The median fluorescent intensity (MFI) values of positively labeled cells were expressed as mean ± SEM of the entire sample, and data was represented as % AD control for all groups (we normalized the data in this way to account for variation in fluorescent intensities between batches).

### Immunohistochemistry

Perfused sections (30 μm) were mounted on Superfrost Plus glass slides (VWR, Radnor, PA,) and stored at −20 °C. The sections were washed in phosphate-buffered saline (PBS) twice followed by two washes in PBS-T (0.05% Triton-X). Then, the sections were incubated with a blocking buffer (2.5% normal horse serum in PBS-T) at room temperature for 60 min. The sections were subsequently incubated overnight at 4 °C with primary antibodies. Primary antibodies for immunohistochemistry were used as follows: goat anti-IBA-1 (1:500; Abcam, Cambridge, MA), rabbit anti-DOR (1:50; Santa Cruz, Billerica, MA), rabbit anti-MOR (1:500; Antibodies Inc., Davis, CA), rabbit anti-GFP (1:2000; Abcam, Cambridge, MA), rabbit anti-ß-endorphin (1:1000, Peninsula Laboratories, Cat#T-4045), and goat anti-POMC (1:400, Santa Cruz, Cat# SC-18262). After the primary antibody incubation and PBS washes, sections were incubated with peroxidase-coupled anti-rabbit (ImmPRESS reagent; Vector Laboratories, Inc., Burlingame, CA) for 3,3′-diaminobenzidine peroxidase (DAB) or Alexa Fluor secondary antibodies (488 and 594; 1:500; Life Technology, Thermo Fisher Scientific, Grand Island, NY) for immunofluorescence. Sections were then mounted with DAPI (Vector Laboratories, Burlingame, CA) and sealed with nail polish. For DAB staining, antigen localization was achieved by using the 3,3′-diaminobenzidine peroxidase reaction (resulting brown staining). After DAB staining, sections were dehydrated in ethanol and mounted in permount (Thermo Fischer Scientific). To evaluate the immunohistochemical staining intensity, animals in each experimental group were photographed using Nikon-TE 2000 inverted microscope (Nikon Instruments Inc., Melville, NY). Pixel density and cell counting were quantified using ImageJ software (National Institutes of Health, Bethesda, MD). To quantify the number of POMC neurons in the arcuate nucleus, serial coronal sections frozen the brains were made using Leica cryostat at 30 μm in thickness from stereotaxic plates 19 to plates 23 (Bregma −2.3 to −4.3 mm) spanning the arcuate nucleus (ARC) area [23]. Every 4th serial section of the arcuate areas of treated animals was collected and was placed on each slide containing one ad libitum, one pair-fed, or alcohol-fed animal brain section. Pictures were taken using C-1 Confocal Nikon-TE 2000 inverted microscope (Nikon Instruments Inc., Melville, NY). Total numbers of POMC and BEP expressing cells were counted in the ARC and presented as percentage of AD control. For 3D analysis of POMC and microglial interactions, confocal images (Zeiss LSM 710; Oberkochen, Germany) were created using a 20× objective and stacked at 1 mm/step, resulting in 10 mm images. 3D interaction analysis between microglia and POMC neurons was performed using Imaris 8.2 (Bitplane, Concord, MA).

### Detection of protein levels by Western blot

Mediobasal hypothalamic tissue samples or microglial cell pellets were used for protein measurements using Western blot procedures. Tissue or cell extracts were processed for protein extraction followed by quantification of total protein levels by Bradford Assay (Bio-Rad Laboratories, Hercules, CA). Protein levels of MOR and DOR were determined by Native-PAGE. Sixty micrograms of protein was loaded for each sample and proteins were resolved using native-PAGE with 10% acrylamide resolving gel. For TNF-α, NF-κB, p38, and IBA-1, Western blot proteins were resolved using SDS-PAGE (Nu-sep, Tris-HEPES; NH-21-420) resolving gel. About 50 μg of total protein was run in 4–20% SDS-PAGE and transferred to nitrocellulose membrane at 30 V overnight at 4 °C. The membranes were blocked in Odyssey Blocking Buffer in PBS (LI-COR Biotechnology, Lincoln, NE) at 4 °C for 5 h or blocked in 5% non-fat dry milk-TBS-0.1% Tween 20 (TBST) at room temperature for 1 h. The membranes were incubated with primary antibody in the same blocking buffer with 0.2% Tween-20 at 4 °C overnight. The primary antibodies used were rabbit anti-Iba-1 (1:400; Wako Pure Chemical Industries, USA), mouse anti-MHC Class II (OX-6) (1:500; Abcam, 55152, Cambridge, MA), rabbit anti-DOR (1:1000; EMD Millipore, Billerica, MA), rabbit anti-MOR (1:5000; EMD Millipore, Billerica, MA), mouse anti-TNF-α (1:1000; Abcam, Cambridge, MA), and mouse anti-actin antibody (1:5000; EMD Millipore, Billerica, MA). Some membranes were washed in PBST (PBS with 0.1% Tween-20) and then incubated with corresponding infrared secondaries (680RD Goat anti-Mouse and 800CW Goat anti-Rabbit IgG, LI-COR Biotechnology, Lincoln, NE) at room temperature for 90 min. The membranes were washed in PBST, and then PBS, and scanned in an Odyssey Infrared Imaging System (LI-COR Biotechnology, Lincoln, NE). Other membranes were washed four times with TBST and then incubated with corresponding peroxidase conjugated secondary antibody (HRP conjugated, 1:1000) at room temperature for 1 h. These membranes were washed six times with TBST and incubated with ECL reagent and were developed on the film by autoradiography. The protein band intensities were determined by Image Studio Lite software (LI-COR Biotechnology, Lincoln, NE) or by ImageJ 1.37v Analysis software (Wayne Rasband, National Institute of Health, USA), and protein expression was normalized with corresponding beta-actin band intensity.

### Quantification of chemokines and cytokines in microglial supernatant

Supernatant of microglial cells treated with agents was analyzed for multiple cytokines and chemokines using Bio-Plex Pro rat cytokine assay (Bio-Rad Laboratories, Hercules, CA). All reagents needed for the assays were provided in the kits. Calibration of the instrument was performed for each use, along with regular monthly recommended system validation, and all samples were assayed in duplicate. Data were obtained using the Bio-Plex Manager software program (Bio-Rad version 4.1.1) for standardization and standard curve acquisition.

### Quantitative reverse transcription polymerase chain reaction (PCR) for gene expression

Gene expression levels of TLR4, MCP-1, and CSFR1 in the hypothalamic tissues or microglial cells were measured by quantitative real-time PCR (SYBR green assay). Total RNA of each mediobasal hypothalamus sample was extracted using RNeasy purification kit (Qiagen, Valencia, CA) and converted to first-strand complementary DNA (cDNA) using high-capacity cDNA reverse transcription kit (Applied Biosystems, Carlsbad, CA). The following primers were used: Forward/Reverse TLR4 (TGCCTCTCTTGCATCTGGCTGG/CTGTCAGTACCAAGGTTGAGAGCTGG), CSFR1 (GCTCGATGTCCTGCTCTGTGA/CCTGCACTCCATCCATGTCA), MCP1 F/R (GGCCTGTTGTTCACAGTTGCT/TCTCACTTGGTTCTGGTCCAGT), and GAPDH F/R (AGACAGCCGCATCTTCTTGT/CTTGCCGTGGGTAGAGTCAT). RT-PCR was performed at 95 °C for 5 min followed by 45 cycles of 95 °C for 30 s, 62 °C for 30 s, and 72 °C for 40 s in Applied Biosystems 7500 Real-time PCR system (ABI, Carlsbad, CA). Relative quantity of mRNA was calculated by relating the PCR threshold cycle obtained from the tested sample to relative standard curves generated from a serial dilution of cDNA prepared from total cDNA and then quantified as a ratio of GAPDH.

### Statistical analysis

Results are expressed as mean ± SEM. One-way ANOVA with the Newman-Keuls post hoc analysis was used to analyze the differences between multiple groups. The value P < 0.05 and onwards was considered significant. Data were analyzed using Prism 5.0 (Graph Pad Software).

## Results

### Alcohol effects on microglia and proopiomelanocortin (POMC) neuron interactions in the hypothalamus during the developmental period

**Fig. 1:**
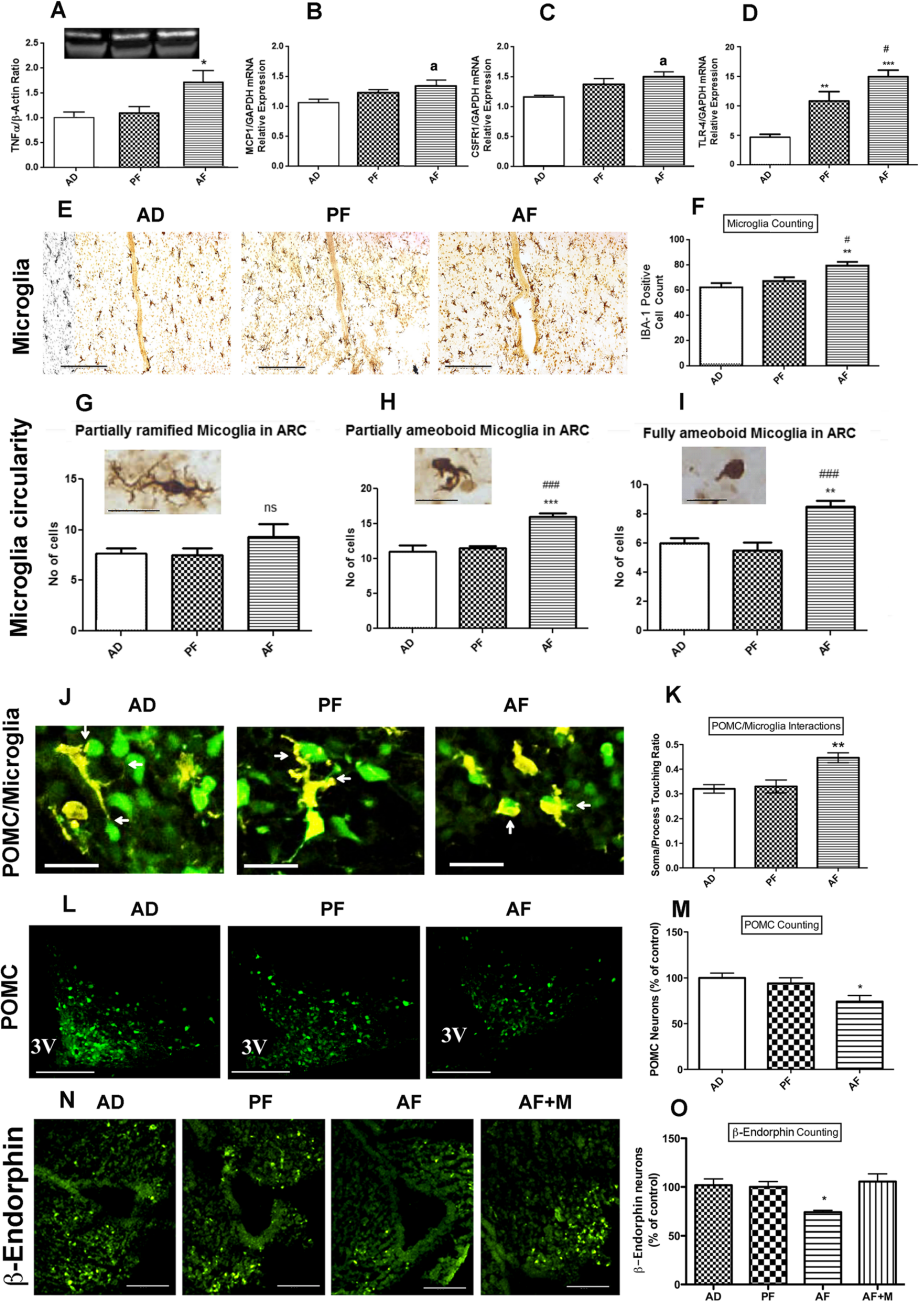
Effect of fetal alcohol exposure on microglia and proopiomelanocortin neuron interaction in the hypothalamus. Showing the changes in the protein level of inflammatory cytokines TNF-α (**a**), mRNA level of cytokine MCP1 (**b**), cytokine receptors CSFR1 (**c**), and TLR4 (**d**) in the mediobasal hypothalamus (MBH) of alcohol-fed (AF), pair-fed (PF) and ad lib-fed rats on postnatal day (PND) 6 as determined through Western blot and q-RTPCR, respectively. Representative photographs of IBA-1-positive cells (**e**) and histograms representing the mean ± SEM number of IBA-1-positive cells (**f**) in the MBH of AF, PF, and AD rats on PND 6. *Scale bars* shown in three photographs of panel **e** are 200 μm/each. Characterization of IBA-1-stained microglial cells in the mediobasal hypothalamus based on circularity (partially ramified (**g**); partially amoeboid (**h**); fully amoeboid (**i**)) in AD, PF, and AF of rat pups on PND 6. *Scale bars* in these figures are 20 μm/each. Representative 3D rendering of IBA-1 microglia and GFP-POMC neurons interacting (**j**).*Scale bars* are 20 μm/each. Quantification of soma/process interaction of microglia with POMC neurons (**k**). Representative images of POMC-stained neurons in the arcuate nucleus (**l**) and histograms representing the mean ± SEM number of POMC-positive cells in the arcuate nucleus of AF, PF, and AD rats on PND 6 (**m**). *Scale bars* are 200 μm/each. Representative images of ß-endorphin-stained neurons in the arcuate nucleus (**n**) and histograms representing the mean ± SEM number of ß-endorphin-positive cells in the arcuate nucleus of AD, PF, and AF and AF + M (minocycline-treated and alcohol-fed) rats on PND 6 (**o**). *Scale bars* are 200 μm/each. Data are represented as mean ± SEM (*n* = 5–7). The differences between AD, PF, and AF were compared by one-way analysis of variance (ANOVA) and the Newman-Keuls posttest. *p < 0.05, **p < 0.01, ***p < 0.001, AF vs PF and AD, ^a^ *p* < 0.05, AF vs AD, ^#^ *p* < 0.05, AF vs PF, ^##^ *p* < 0.01, AF vs PF, ^###^ *p* < 0.001, AF vs PF

Previously, we have shown that alcohol exposure during the developmental period reduces POMC/ß-EP cell number by increasing this neuronal apoptotic death in the hypothalamus [8, 10]. Using the rat model of neonatal alcohol feeding (equivalent to third trimester binge human alcohol use) which elevates blood level of alcohol about 150–200 mg/dl and increases POMC/ß-EP neuronal apoptotic death [10], we determined the changes in the levels of pro-inflammatory signal molecules in the mediobasal hypothalamus where most of the POMC neurons are distributed. We measured some of the important pro-inflammatory signal molecules (TNF-α, MCP1, CSFR1, and TLR4), which are known to promote neuronal death [24]. As shown in Fig. 1, protein levels of TNF-α (Fig. 1a) and mRNA levels of MCP1 (Fig. 1b) were elevated in the hypothalamus of alcohol-fed (AF) animals as compared to control-fed animals. Similarly, mRNA levels of TLR4, an innate immune system activating receptor, and CSFR1, a cytokine receptor, were elevated in the hypothalamus of AF rats as compared to controls (Fig. 1c, d).

In the CNS, microglia are host CNS immune cells and are known to participate in the cytotoxic response, immune regulation, and injury resolution. [3]. We therefore determined the number of microglia within the hypothalamus by counting the cells stained with IBA-1, a calcium-binding protein that is specifically expressed in microglia in the brain [25]. As shown in Fig. 1e, f the number of IBA-1-stained microglia was higher in the mediobasal hypothalamus in AF rats as compared to those in PF and AD control rats.

Microglia can be categorized based on their circularity (process length) into different stages of activation ranging from ramified microglia (resting microglia) to amoeboid microglia (activated microglia). Microglial cells were counted based on morphology in IBA-1 immunostained sections of AD, PF, and AF rat pups’ mediobasal hypothalamus and data are shown Fig. 1g–i. These data show a significant increase in the number of partially amoeboid and fully amoeboid microglia in the hypothalamus of alcohol fed as compared to AD and PF rats.

It is not known how microglia-POMC neuron interactions are modified at the cellular level by early life ethanol exposure. Our initial investigation on microglia-POMC neuron interactions using double-staining immunohistochemistry procedures in rat hypothalamic tissue did not produce satisfactory outcomes due to fractionated staining of the POMC protein within the cell. However, when we employed offspring from adult transgenic mice (C57BL/6J) expressing the fluorescent protein EGFP in POMC neurons and employed immunocytochemical staining for GFP, we were able to visualize the whole POMC cell mass (Fig. 1j). Additionally, uses of double-staining immunohistochemistry (anti-GFP and anti-IBA-1) and 3D analysis of confocal imaging were able to identify strong POMC and microglial interactions. Neonatal alcohol treatment in mice promoted microglial soma interaction but reduced microglial process interactions with POMC neurons (Fig. 1j, k) and reduced the number of POMC neurons (Fig. 1l, m). Suppression of microglial activation during the ethanol feeding by minocycline, an inhibitor of microglia, prevented ethanol’s ability to reduce BEP (POMC’s cleavage product) cell number in the hypothalamus of neonatal rats (Fig. 1n, o).

### Role of μ-opioid and δ-opioid receptors in ethanol activation of microglia in the hypothalamus

**Fig. 2:**
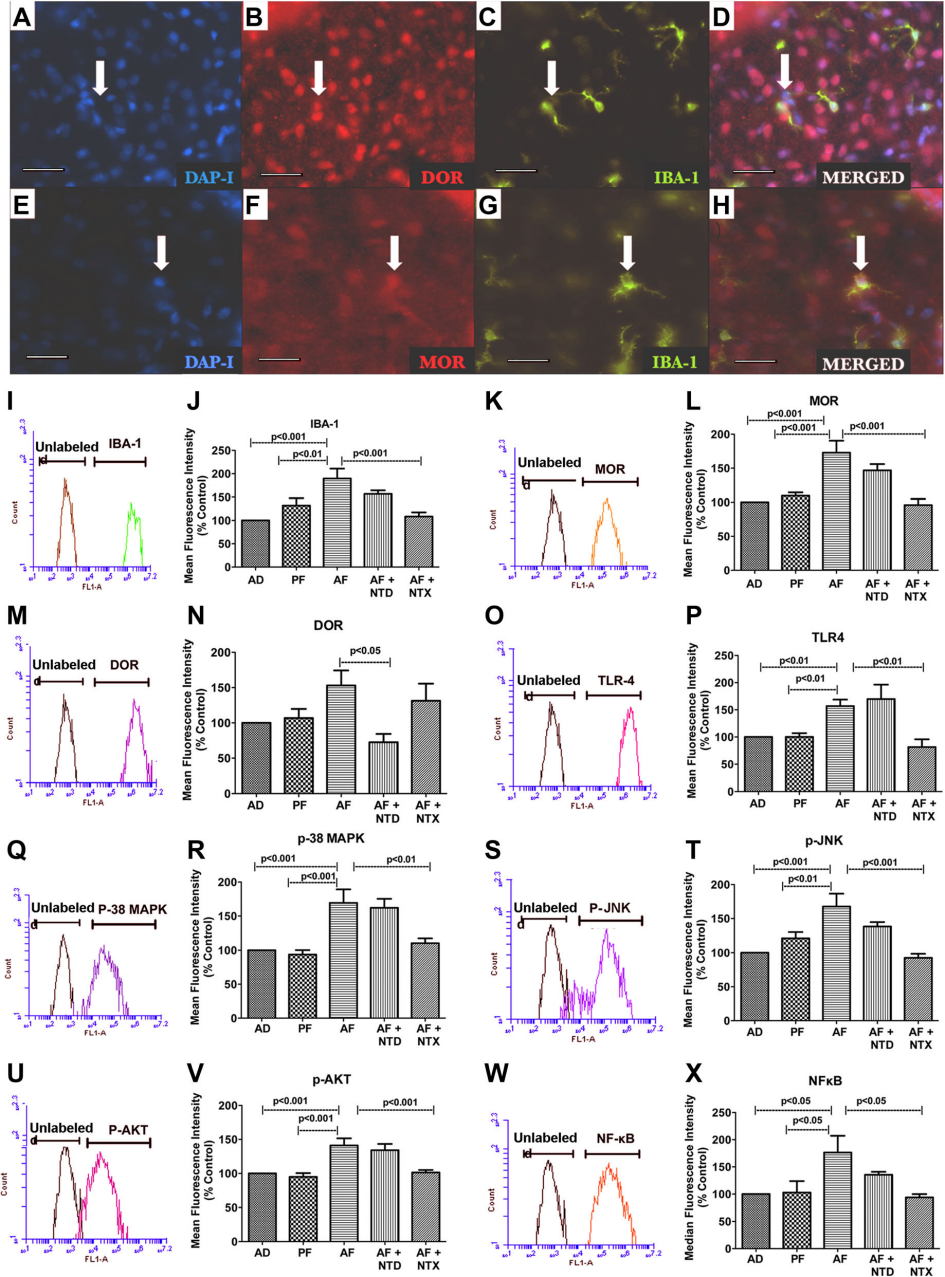
Neonatal alcohol effects on microglial opioid receptors and TLR4 pathway proteins in mediobasal hypothalamus in vivo. Immunohistochemical characterization of double-labeled IBA-1 and MOR/DOR-positive microglia in neonatal pups. Nuclear stain DAPI (**a**, **e**), DOR (**b**), MOR (**f**), and IBA-1(**c**, **g**) immunolabeled cells. Representative merged photographs demonstrate colocalization of DOR/IBA-1 (**d**) and MOR/IBA-1 (**h**). *Scale bars* are 200 μm/each and *arrows* depict the same cell. **i**–**x** showing protein quantification in isolated microglia from ad libitum (AD), pair-fed (PF), alcohol-fed (AF), alcohol-fed and naltrindole-treated (AF + NTD), and alcohol-fed and naltrexone-treated (AF + NTX) neonatal rat pups at PND 6 by flow cytometry. Histograms show representative staining of isolated microglia for each protein compared to unstained or unlabeled control cells. Bar graphs show mean or median fluorescence intensity of staining of IBA-1 (**i**, **l**), MOR (**k**, **l**), DOR (**m**, **n**), TLR4 (**o**, **p**), p-38 MAPK (**q**, **r**), p-JNK (**s**, **t**), p-AKT (**u**, **v**), and NF-κB (**w**, **x**) from microglia isolated from each treatment group. Data are represented as mean ± SEM (*n* = 7–11). Data were compared by one-way analysis of variance (ANOVA) and the Newman-Keuls posttest. Differences between control and other treatment groups or ethanol and ethanol and opioidergic drug treatment groups are shown by *lines with p values* on the *top* of bar graphs

Whether opioid receptors MOR and DOR participate in the communication between POMC neurons and microglia within the hypothalamus during ethanol-induced neurotoxicity was tested. First, we conducted a colocalization study of MOR and DOR on microglia within the hypothalamus using double immunohistochemistry methods. Figure 2a–h shows colocalization of MOR- and DOR-labeled cells with IBA-1-positive cells, showing that microglia cells do express MOR and DOR proteins.

To determine the changes in levels of MOR and DOR in microglia and the mediatory role of these receptors in activation of microglia by neonatal alcohol, we isolated microglia by differential gradient centrifugation using OptiPrep gradient from hypothalamic tissues of neonatal rats (PND 6, both sexes) fed with control diets or alcohol diet and treated with or without a DOR antagonist naltrindole (NTD) or a MOR antagonist naltrexone (NTX). We found that differential gradient centrifugation method was able to isolate microglia with >90% purity, as determined by the IBA-1 positivity in flow cytometry. A preliminary study was conducted to determine if a sex difference exists in ethanol actions on microglia. We found that the effects of alcohol show similar trends in the effects of treatment that was present in both males and females. We therefore conducted all studies using neonates of both sexes (see Additional file 1: Figure S1). Measurements of IBA-1-positive cells by flow cytometry revealed that neonatal ethanol treatment, as observed by immunocytochemical localization of IBA-1 positive cells in the hypothalamus (Fig. 1e, f), increased IBA-1 protein, showing upregulation of this protein after AF treatments (Fig. 2i). The alcohol-induced increase in IBA-1 is reversed by the treatment of MOR antagonist NTX but not by DOR antagonist NTD (Fig. 2i). The level of MOR protein in microglia was increased after alcohol treatment. Naltrexone, but not naltrindole, blocked this action of ethanol on MOR protein (Fig. 2j). Alcohol treatment increased the mean level of DOR protein in microglia, but this trend was not significant. Naltrindole, but not naltrexone, reduced the level of DOR protein in microglia. We also found that neonatal ethanol increased a pro-inflammatory signal receptor (TLR4; Fig. 2l) and pro-inflammatory cell signaling molecules (p-38 MAPK, M; p-JNK, N; p-AKT, O; and NF-κB, P). In each case, naltrexone prevented ethanol effects on pro-inflammatory cell signaling molecules in microglia, but naltrindole failed to alter ethanol effects. These data suggest that the microglial cell population within the hypothalamus expresses MOR and DOR , and their expression levels change following ethanol challenges. In addition, ethanol activates microglia MOR expression, which is accompanied by increased levels of pro-inflammatory cell signaling molecules in microglia.

### Effects of μ-opioid receptor and δ-opioid receptor agonists on ethanol’s actions on microglial cellular proteins in primary cultures

**Fig. 3:**
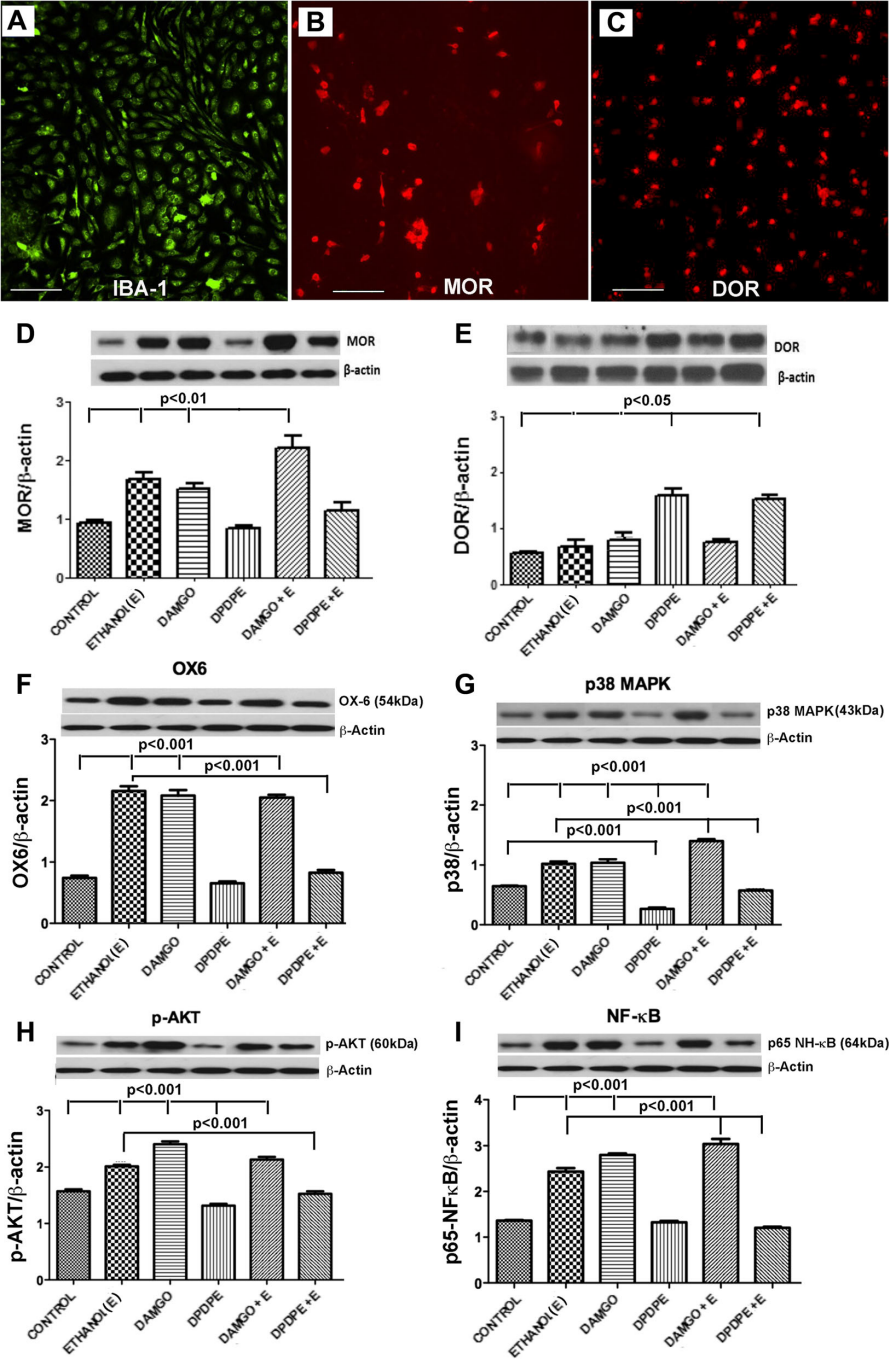
In vitro effects of ethanol and opioid receptor agonists on MOR and DOR and TLR4 pathway protein levels in hypothalamic microglia. Representative photographs of immunostaining of **a** IBA-1-, **b** MOR-, and **c** DOR-positive cells in microglia maintained in primary cultures. *Scale bars* are 200 μm/each. Bar graphs are showing the effects of ethanol (50 mM) with or without MOR agonist (DAMGO, 50 μM) or DOR agonist (DPDPE, 10 nM) on protein levels of MOR (**d**), DOR (**e**), OX6 (**f**), p38 MAPK (**g**), p-AKT (**h**), NF-κB (**i**). The protein levels were measured by Western blot. Treatment time was 24 h. Data are represented as mean ± SEM (*n* = 6–8). Data of all groups were compared by one-way analysis of variance (ANOVA) and the Newman-Keuls posttest. Differences between control and other treatment groups or ethanol and ethanol and opioidergic drug treatment groups are shown by *lines with p values* on the *top* of bar graphs

In order to gain further knowledge on the role of opioid receptors in ethanol-controlled microglial cell function, we first investigated the interaction between ethanol and opioid receptors on microglia in an isolated culture condition. Microglial cells isolated from the neonatal rat hypothalamus and grown in primary cultures, when stained for MOR and DOR protein by immunohistochemistry, showed detectable amounts of both MOR and DOR proteins (Fig. 3a–c). When microglia cells were challenged with vehicle control, ethanol, MOR agonist DAMGO, or DOR agonist DPDPE alone or with ethanol, these cells showed differential activation response. As shown in Fig. 3d, both ethanol and DAMGO alone or together increased MOR protein levels, as compared to controls. DPDPE treatment alone and together with ethanol increased DOR protein levels (Fig. 3e). Treatments with ethanol or DAMGO alone activated microglia, as detected by increased microglial activation marker protein OX6 (Fig. 3f). DPDPE alone did not produce any effect on the OX6 level but significantly affected ethanol’s ability to increase OX6 levels. DAMGO did not affect ethanol’s ability to increase OX6 levels. These data suggest that MOR and DOR differentially regulate ethanol activation of microglia. We also studied the changes in the pro-inflammatory cell signaling molecules in microglia in cultures following ethanol and opioid receptor antagonist treatments. In the case of p38 MAPK signaling, ethanol and DAMGO increased p38 MAPK levels, while DPDPE decreased this signaling protein’s levels. When combined, DAMGO potentiated ethanol’s action and DPDPE decreased ethanol’s action on p38 MAPK levels in microglia (Fig. 3g). In the cases of p-AKT and NF-κB, ethanol and DAMGO alone also increased p38 MAPK levels, and when combined with ethanol, it produced similar levels of p-AKT but elevated levels of NF-κB. DPDPE alone did not change the levels of p-AKT and NF-κB proteins, but when combined with ethanol, it decreased ethanol-induced changes in p-AKT and NF-κB protein levels in microglia (Fig. 3h, i). Together, these in vitro data confirm the in vivo findings that hypothalamic microglia express MOR and DOR proteins, and they function differently to activate or suppress pro-inflammatory signaling molecules in microglia in the presence of ethanol.

### Effects of μ-opioid receptor and δ-opioid receptor agonists on ethanol-induced secretion of pro-inflammatory and anti-inflammatory cytokines from microglia in primary cultures

**Fig. 4:**
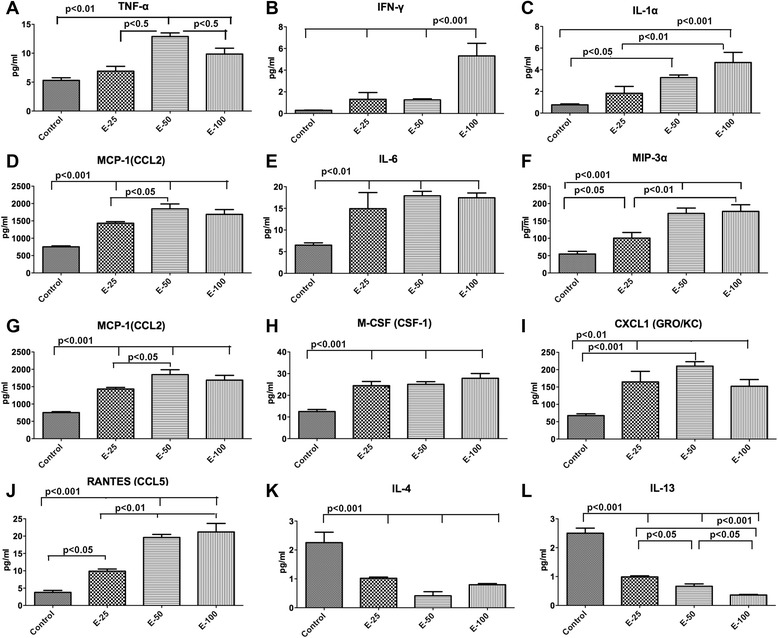
In vitro dose-response effects of ethanol on cytokine secretion from microglia in primary cultures. Showing the changes in the protein levels of pro-inflammatory cytokines TNF-α (**a**), IFN-γ (**b**), IL-1α (**c**), L-1β (**d**), IL-6 (**e**), MIP-3α (**f**), MCP-1 (**g**), M-CSF (**h**), CXCL1 (**i**), and RANTES (**j**) and anti-inflammatory cytokines IL-4 (**k**) and IL-13 (**l**) in microglial conditioned medium following 24-h treatment with various doses of ethanol (25, 50, 100 mM). Data are represented as mean ± SEM (*n* = 6). Data of all groups were compared by one-way analysis of variance (ANOVA) and the Newman-Keuls posttest. Differences between groups are shown by *lines with p values* on the *top* of bar graphs

**Fig. 5:**
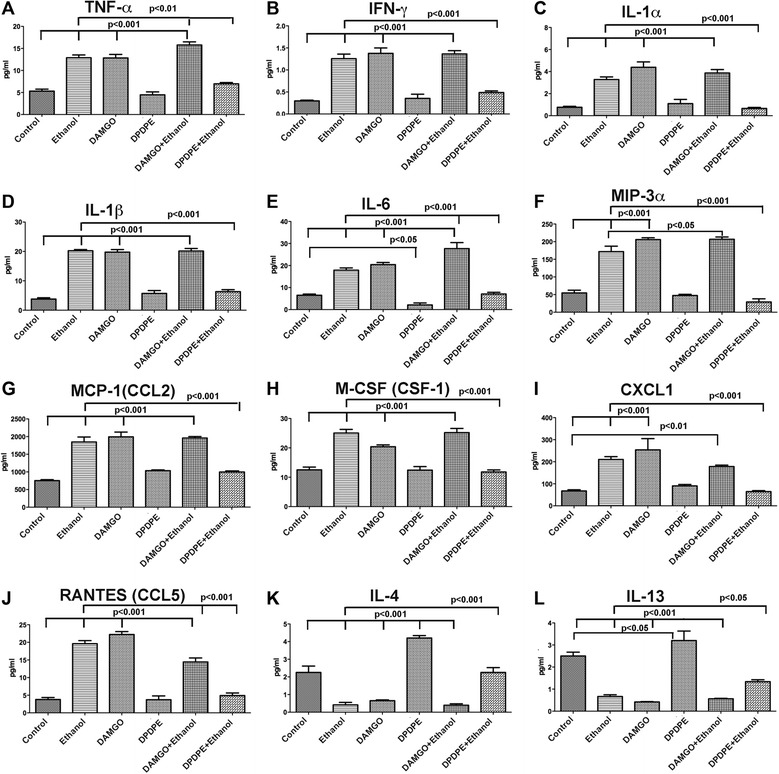
In vitro effects of ethanol with or without opioid receptor agonists on cytokine secretion from hypothalamic microglia in primary cultures. Showing the changes in the protein levels of pro-inflammatory cytokines TNF-α (**a**), IFN-γ (**b**), IL-1α (**c**), IL-1β (**d**), IL-6 (**e**), MIP-3α (**f**), MCP-1 (**g**), M-CSF (**h**), CXCL1 (**i**), and RANTES (**j**) and anti-inflammatory cytokines IL-4 (**k**) and IL-13 (**l**) in microglial conditioned medium following 24-h treatment with ethanol (50 mM) with or without MOR agonist (DAMGO, 50 μM) or DOR agonist (DPDPE, 10 nM). Data are represented as mean ± SEM (*n* = 6). Data were compared by one-way analysis of variance (ANOVA) and the Newman-Keuls posttest. Differences between control and other treatment groups or ethanol and ethanol and opioidergic drug groups are shown by *lines with p values* on the *top* of bar graphs

We employed multiplex ELISA and primary cultures of hypothalamic microglia in order to characterize secretion patterns of all major cytokines by microglia following an ethanol challenge in the presence and absence of opioid receptor agonists. In the initial dose-response study employing various doses of ethanol (25–100 mM), we identified that a 50 mM dose of ethanol maximally increased the levels of ten pro-inflammatory cytokines (TNF-α, IFN-γ, IL-1α, IL-1β, IL-6, MIP-3α, MCP-1, M-CSF, CXCL1, chemokine (C-C motif) ligand 5 (RANTES)) and reduced two anti-inflammatory cytokines (IL-4 and IL-13) as compared to the control group (Fig. 4). The cytokine responses to 50 mM dose of ethanol were compared in the presence and absence of MOR or DOR agonists (Fig. 5). Like ethanol, the MOR agonist DAMGO increased the levels of all ten pro-inflammatory cytokines and reduced the two anti-inflammatory cytokines. When combined, DAMGO was not able to change the levels of most of the pro-inflammatory and anti-inflammatory cytokines induced by ethanol, with exception of MIP-3α which was moderately elevated (Fig. 5f) and RANTES, which was moderately decreased (Fig. 5j). The DOR agonist DPDPE alone did not change the basal levels of pro-inflammatory cytokines (Fig. 5a–j) but increased the basal levels of anti-inflammatory cytokines (Fig. 5k, l). DPDPE also prevented ethanol’s stimulatory actions on pro-inflammatory cytokines and ethanol’s inhibitory actions on anti-inflammatory cytokines. These results support the concept that MOR and DOR function differently in hypothalamic microglia and identify a pro-inflammatory function for MOR and anti-inflammatory function for DOR.

### Role of μ-opioid and δ-opioid receptors on microglia in ethanol-induced POMC neuronal apoptosis

**Fig. 6:**
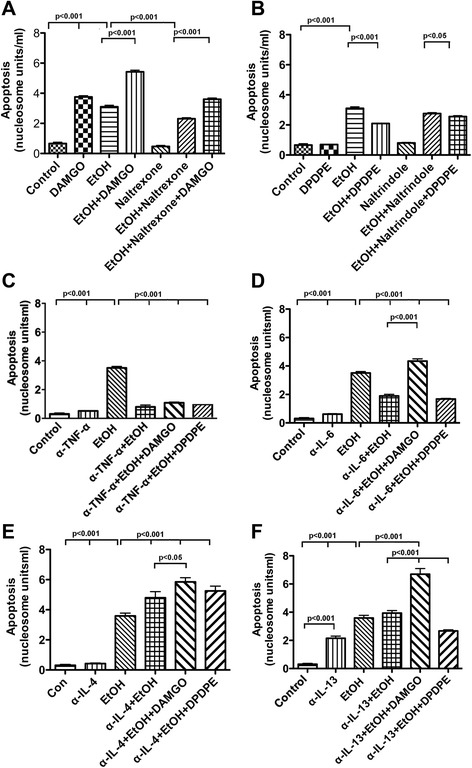
The effect of opioid agonists and antagonists and immunoneutralization of inflammatory cytokines TNF-α and IL-6 on the ability of ethanol-or opioid-activated microglial conditioned media to induce apoptosis of POMC neurons. POMC neurons were differentiated from neural stem cells in culture and maintained in T25 flasks (1 × 10^6^/well) for 2 days and then treated for 24 h with microglial conditioned media exposed to opioidergic agents for 24 h before determining neuronal apoptosis using a nucleosome assay. Bar graphs are showing the apoptotic effects of ethanol (50 mM) with or without DAMGO (50 μM), naltrexone (10 ng/ml), or DAMGO and naltrexone (**a**); ethanol (50 mM) with or without DPDPE (10 nM), naltrindole (50 μM), or DPDPE and naltrindole (**b**). Microglial conditioned media from ethanol with or without opioidergic agonist-treated cultures were mixed with antibody to TNF-α (1 ng/ml (**c**)), antibody to IL-6 (0.5 ng/ml (**d**)), antibody to IL-4 (1 ng/ml (**e**)), or antibody to IL-13 (5 ng/ml (**f**)) and added to POMC neuron cultures for 24 h to determine neuronal apoptosis. The effects of immunoneutralization of inflammatory and anti-inflammatory cytokines on ethanol with or without opioid-activated POMC neuronal apoptosis are shown in bar graphs. Each *bar* represents mean ± SEM of 5–8 samples. Data were compared by one-way analysis of variance (ANOVA) and the Newman-Keuls posttest. Differences between groups are shown by *lines with p values* on the *top* of bar graphs

In order to evaluate if opioid receptors on microglia participate in ethanol neurotoxic action on POMC neurons, we treated microglial cell cultures with ethanol with or without MOR or DOR agonist or antagonist and then microglial conditioned media were removed and added to POMC cell cultures to conduct the apoptotic studies. Data shown in Fig. 6a indicate that the conditioned medium of microglial cells treated with the MOR agonist DAMGO or with ethanol (EtOH) increased nucleosome levels in POMC neuronal cells in a similar magnitude. However, conditioned medium of microglial cells treated with both DAMGO and ethanol increased nucleosome levels in POMC cells more than those produced by ethanol alone. The conditioned medium from MOR antagonist naltrexone-treated microglia had no effect on nucleosome levels. The conditioned medium of microglia treated with both naltrexone and ethanol increased nucleosome levels in POMC cells much less than those produced by ethanol alone. Naltrexone co-treatment also reduced the ability of ethanol to increase nucleosome levels in POMC cells. Figure 6b shows that DOR agonist DPDPE or antagonist naltrindole alone treated microglia culture media produced no significant effects on the basal level of nucleosomes in POMC cells. However, the conditioned medium of microglia treated with both ethanol and DPDPE, but not with ethanol and naltrindole, had lower levels of nucleosomes in POMC cells than those produced by ethanol alone. Naltrindole co-treatment also reduced the ability of ethanol and DPDPE to increase nucleosome levels in POMC cells. These data support a mediatory role of MOR in ethanol-activated microglia killing of POMC neurons. Also, these data identify a protective effect of the DOR agonist in ethanol-activated microglia killing of POMC neurons.

We investigated if pro-inflammatory or anti-inflammatory cytokines are involved in inducing POMC neuronal apoptosis following microglial activation by ethanol with or without a MOR agonist DAMGO or a DOR agonist DPDPE. The immunoneutralization procedure was undertaken to block two pro-inflammatory cytokines TNF-α and IL-6 which are shown to be produced by microglia in elevated amount following MOR and ethanol treatment (Figs. 4 and 5) and are known to be activated during TLR4-mediated neuroinflammation [26]. We also used immunoneutralization procedures to block two anti-inflammatory cytokines IL-4 and IL-13 which are produced in elevated amount from microglia following DOR activation (Figs. 4 and 5). Figure 6c shows that TNF-α antibody alone treatment did not change the ability of microglial conditioned medium to increase nucleosome levels in POMC neurons. Co-treatment of DAMGO or DPDPE with ethanol failed to alter the nucleosome suppressive effect of TNF-α antibody (6C). Similarly, IL-6 antibody alone did not change the nucleosome activity in POMC neurons but reduced, at a lesser degree, the ability of ethanol-treated microglial conditioned medium to stimulate nucleosome activity in POMC neurons (Fig. 6d). Co-treatment of DAMGO, but not DPDPE, with ethanol prevented the nucleosome suppressive effect of IL-6 antibody (6D). In contrast, antibodies against the anti-inflammatory cytokines IL-4 stimulated the ability of ethanol-alone-treated microglial conditioned medium to stimulate nucleosome activity in POMC neurons. Co-treatment of DAMGO with ethanol, but not DPDPE with ethanol, enhanced the nucleosome stimulatory effect of IL-4 antibody (6E). Antibodies against IL-13 increased the basal level of nucleosome in POMC neurons and only moderately, but not significantly, increased ethanol-induced nucleosome level in POMC neurons. Furthermore, co-treatment of DAMGO with ethanol enhanced, while co-treatment of DPDPE with ethanol reduced the nucleosome stimulatory effect of IL-4 antibody (6F). These data suggest that MOR and DOR on microglia differentially modulate pro-inflammatory and anti-inflammatory signaling molecules to regulate ethanol’s apoptotic effect on POMC neurons.

## Discussion

Fetal alcohol exposure increases POMC neuronal death in the hypothalamus [10] and results in abnormality in the feedback regulation of stress axis function during a stress challenge [14, 15, 27, 28], but the mechanism by which POMC neuronal death occurs is not completely understood. In this study, using a rat animal model, we demonstrated that ethanol exposures during the neonatal period, a period equivalent to third trimester of human pregnancy, increase the levels of proinflammatory signaling molecules within the hypothalamus, an area where many POMC neurons are distributed. In addition, we demonstrated an increased number of activated amoeboid microglia (reactive microglia) in the hypothalamus. Activation of microglia towards the amoeboid morphology is known to be associated with inflammatory response, increased cytotoxicity, as well as phagocytosis [1]. Additionally, we showed enhanced microglial contact with POMC cell soma and decreased hypothalamic POMC cell number, which was reversed by the co-treatment with a microglial activation blocker minocycline. Although minocycline is not a selective microglia blocker [29], it has a significant inhibitory effect on the inflammatory phase of microglia [30]. We have used minocycline to determine its ability to block microglia-mediated ethanol action on POMC cells in vivo (Fig. 1n, o). The mediatory role of microglia in ethanol neurotoxic action is consistent with the data of our current (Fig. 6) and our previous in vitro studies [7–9]. We have also observed greater microglial soma interaction with POMC neurons following alcohol feeding, which strongly supports interaction between these two cell types during alcohol exposures. Together, these data suggest that communication between microglia and POMC neurons is important in the establishment of ethanol’s neurotoxic effect on POMC neurons in the hypothalamus during the developmental period.

Recently, it has been shown that the communication between neurons and microglia involves several immunomodulatory factors and signaling molecules including neurotransmitters and their receptors [20]. POMC neurons produce opioid peptides [31], and therefore, opioid receptors on microglia may be potential target molecules. Our data provide evidence that a significant level of MOR and DOR proteins is expressed in microglial cells in the hypothalamus and their levels are differentially modulated by ethanol. The presence of MOR in microglia has been previously demonstrated by immunocytochemistry, Western blot, and PCR detections in the CNS [31]. Some studies detected DOR proteins by immunocytochemistry and RT-PCR in primary cultures of forebrain microglial cells [32], but others failed to detect DOR proteins by a similar technique in primary microglial cultures from the cortical area of the brain [33]. We detected both MOR and DOR proteins in microglia derived from the hypothalamus using both immunocytochemistry and flow cytometry methods. The difference between our and Mika et al. [33] findings might be related to differential characteristics of microglia in various parts of the brain [34].

The question that arises is what are the physiological ligands for MOR and DOR in ethanol-induced microglial activation and inflammation. Our current study did not investigate the endogenous ligands for MOR and DOR in ethanol-induced microglial activation. However, we could postulate from the published data that BEP cleavage products may play a role in differential activation of MOR and DOR in microglia. This possibility arises since alcohol consumption and inflammation are known to alter endopeptidase activity in the brain [35, 36] and the production of β-endorphin (BEP) fragments (e.g., BE 1–17, BE 1-18, BE 1-19, BE 20–31; [37, 38]). Additionally, it has been shown that smaller N-terminal BEP fragments produce higher efficacy to DOR while all BEP fragments produce similar efficacy to MOR [36]. Additional studies are needed to address this issue.

How ethanol differentially regulates MOR and DOR expression in the microglia? Our data suggest that MOR may be preferentially increased while DOR is not significantly increased on microglia via ethanol, and this may bias the effects of alcohol on the MOR towards a pro-inflammatory M1 effect. We are not certain how ethanol preferentially activate MOR in microglia. One possibility is that ethanol may directly act on microglia to activate MOR. Ethanol may also change endogeneous ligands in the brain to activate MOR in microglia. Further studies are needed to address this issue.

Microglia are able to recognize harmful stimuli and respond by producing inflammatory cytokines such as TNFα, IL-6, IL-1β, IFN-γ, and several chemokines [39]. This cytokine production is essential for the polarization of microglia into what has been termed a classically activated, “M1” state [40]. Division of M2 cells is based on observations that stimulation with various cytokines (e.g., IL-4, IL-13) yields different sets of receptor profiles, cytokine production, chemokine secretion, and function (suppression of inflammation) [41]. Microglial activation and inflammatory molecule expression as a result of ethanol treatment have been well studied during brain development [12, 42, 43]. It has been shown that high doses of ethanol during the developmental period activate microglia to a pro-inflammatory stage and also increase the expression of neuroinflammatory cytokines and chemokines in diverse regions of the brain. Using both in vivo and in vitro model systems, we showed here that a MOR antagonist, but not a DOR antagonist, prevented alcohol activation of microglia and its production of inflammatory cell signaling molecules in the hypothalamus. Additionally, ethanol and a MOR agonist increased the production of microglial activation markers and inflammatory signaling molecules, while a DOR agonist suppressed alcohol activation of microglia and its production of inflammatory cell signaling molecules in cell cultures. Furthermore, ethanol and a MOR agonist increased secretion of pro-inflammatory cytokines but decreased secretion of anti-inflammatory cytokines, while a DOR agonist decreased secretion of pro-inflammatory cytokines but increased secretion of anti-inflammatory cytokines from microglia. We also showed, using an in vitro cell culture model, that ethanol’s apoptotic effect on POMC neurons is mediated by enhanced pro-inflammatory cytokines like TNF-α and IL-6 produced from microglia, and this effect is promoted by a MOR agonist and suppressed by a MOR antagonist or a DOR agonist. In contrast, ethanol’s apoptotic action on POMC neurons is prevented by DOR-activated production of IL-4 and IL-13. Together, these data suggest that opioid receptors MOR and DOR differentially respond to the ethanol challenge and differentially control the production of inflammatory and anti-inflammatory cytokines from microglia to control POMC neuronal apoptosis.

The pro-inflammatory effect of MOR in microglia within the hypothalamus we observed in this study is consistent with the previous findings that morphine and a MOR receptor agonist DAMGO induce the production of pro-inflammatory signaling molecules (e.g., AKT, NF-κB, MAPK) and increase secretion of pro-inflammatory cytokines (e.g., IL-1b, TNF-α, and IL-6), through Akt and MAPK signaling from forebrain microglia [43]. Our data are also in agreement with the findings that MOR deficiency can protect against the neuroimmune response in the CNS to ethanol drinking in rats [44]. Our findings that a DOR agonist induces anti-inflammatory cytokine production and secretion from microglia and prevents ethanol’s apoptotic effects on POMC neurons are also novel and interesting. In this regard, supporting the DOR neuroprotective role is the evidence that DOR activation reversed the hypoxia-induced reduction in BDNF-TrkB signaling and TNF-α secretion in the cortex of hypoxic rats [45] and produced neuroprotective effects in global cerebral ischemic injury and hypoxic neuronal injury [46, 47]. Furthermore, it has been shown that, in contrast to MOR activation, DOR activation in the ventral tegmental area protects against elevated alcohol consumption in rat animal models [48].

## Conclusions

**Fig. 7:**
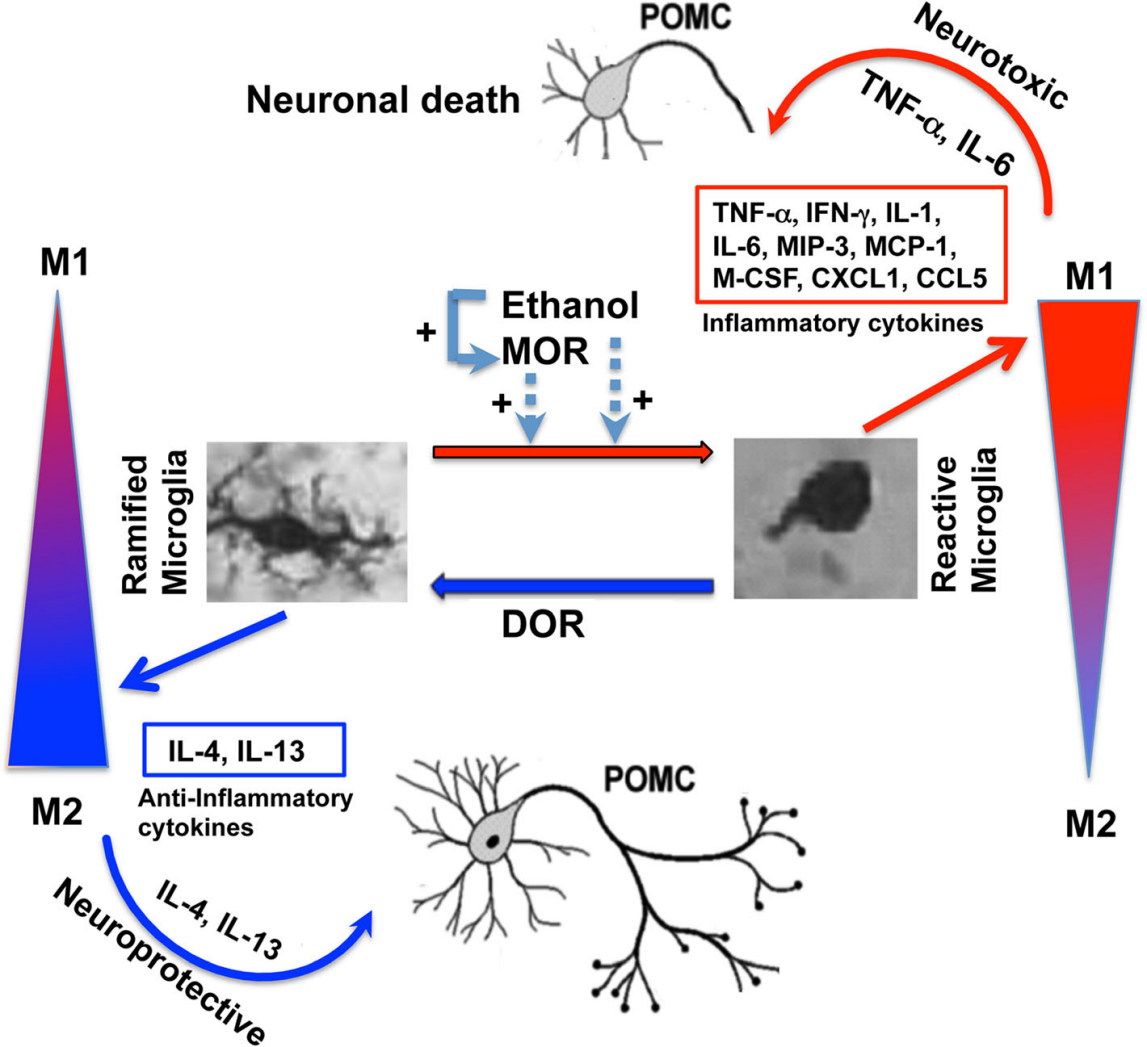
Schematic diagram illustrating the proposed mechanism by which ethanol interacts with opioid receptors to control POMC neuronal apoptosis in the hypothalamus. Ethanol activation of MOR induces microglial polarization towards the M1 phenotype to produce overabundance of inflammatory cytokines and inflammation. Chronic inflammation and overproduction of TNF-α and IL-6 cytokines are cytotoxic to POMC producing neurons. DOR activation increases production of anti-inflammatory cytokines and helps microglial polarization towards the M2 phenotypes. Elevated productions of IL-4 and IL-13 cytokines from M2 microglia reduce ethanol’s ability to increase POMC neuronal apoptosis. DOR agonist may have therapeutic potential to prevent ethanol neurotoxic action

In summary, these data provide strong evidence that alcohol neurotoxic action on POMC neurons results from differential expression of MOR and DOR to promote MOR-activated neuroinflammatory signaling and to reduce DOR-regulated anti-inflammatory signaling in microglia (Fig. 7). Our demonstration of the role of activated microglia as well as the protective effect of a DOR agonist in neuronal apoptosis may present a future approach for understanding fetal alcohol spectrum diseases. Towards that aim, it will be necessary to identify how two similar members of the opioid receptor family with high affinity for endogenous opioid peptides produce differential effects on neuroinflammation. Our data also suggest the possibility that treatment of a DOR agonist with or without a MOR antagonist may provide therapeutic value in prevention of alcohol neurotoxic action on the brain during the developmental period, particularly, the stress axis abnormalities in fetal alcohol exposed patients.

## Additional file

Additional file 1: Figure S1. Neonatal alcohol effects on microglial opioid receptors and TLR4 pathway proteins in the mediobasal hypothalamus of male and female neonates. Showing protein quantification in isolated microglia from ad libitum (AD), pair-fed (PF), alcohol-fed (AF), alcohol-fed and naltrindole-treated (AF + NTD), and alcohol-fed and naltrexone-treated (AF + NTX) neonatal male and female rat pups at PND 6 by flow cytometry. Bar graphs show mean or median fluorescence intensity of staining of IBA-1 (A, B), MOR (C, D), DOR (E, F), TLR4 (G, H), p-38 MAPK (I, J), p-JNK (K, L), p-AKT (M, N), and NF-κB (O, P) from microglia isolated from each treatment group from male and female neonates. Data are represented as mean ± SEM (*n* = 3–7) and were compared by one-way analysis of variance (ANOVA) and the Newman-Keuls posttest. Differences between groups are shown by lines with *p* values on the top of bar graphs. (DOCX 964 kb)

## Abbreviations

AD: Ad libitum fed
AF: Alcohol fed
ANOVA: Analysis of variance
BDNF: Brain-derived neurotrophic factor
BEP: beta-Endorphin
cAMP: Cyclic adenosine monophosphate
CCXL1: Chemokine (C-X-C motif) ligand 1
cDNA: Complementary DNA
CNS: Central nervous system
CSFR1: Colony-stimulating factor 1 receptor
DAB: 3,3′-Diaminobenzidine peroxidase
DAMGO: [D-Ala 2, N-MePhe 4, Gly-ol]-enkephalin
DOR: Delta-opioid receptors
DPDPE: [D-Pen2,5]Enkephalin
FASD: Fetal alcohol spectrum disorders
FBS: Fetal bovine serum
FCS: Fetal calf serum
FITC: Fluorescein isothiocyanate
GFAP: Glial fibrillary acidic protein
HBSS: Hank’s balanced salt solution
HRP: Horseradish peroxidase
IBA-1: Ionized calcium-binding adaptor molecule 1
IFN-γ: Interferon gamma
IL: Interleukin
JNK: c-Jun N-terminal kinases
MAP2: Microtubule-associated protein 2
MAPK: Mitogen-activated protein kinase
MCP-1: Monocyte Chemoattractant Protein 1
MFI: Median fluorescent intensity
MHC: Major histocompatibility complex
MOPS: 3-(N morpholino)
MOR: mu-Opioid receptors
NaCl: Sodium chloride
NF-κB: Nuclear factor kappa beta
NTD: Naltrindole
NTX: Naltrexone
PACAP: Pituitary adenylate cyclase-activating polypeptide
PAGE: Polyacrylamide gel electrophoresis
PBS: Phosphate-buffered saline
PBST: Phosphate-buffered saline with Twin 20
PF: Pair fed
PND: Postnatal day
POMC: Proopiomelanocortin
PPAR-γ: Peroxisome proliferator-activated receptor-γ
RANTES: Chemokine (C-C motif) ligand 5
RBC: Red blood cells
RT-PCR: Reverse transcription polymerase chain reaction; SYBR green
TBST: Tris-buffered saline with Tween 20
TLR4: Toll-like receptor 4
TNF-a: Tumor necrosis factor alpha
TrkB: Tropomyosin receptor kinase B
