# Supplementary material for: Mu-opioid receptor and delta-opioid receptor differentially regulate microglial inflammatory response to control proopiomelanocortin neuronal apoptosis in the hypothalamus: effects of neonatal alcohol
Source: J Neuroinflammation. 2017 Apr 14;14:83. doi: 10.1186/s12974-017-0844-3 (PMC5391607; doi:10.1186/s12974-017-0844-3)

**Additional file 1: Figure S1**. *Neonatal alcohol effects on microglial opioid receptors and TLR4 pathway proteins in the mediobasal hypothalamus of male and female neonates.* Showing protein quantification in isolated microglia from ad libitum (AD), pair fed (PF), alcohol fed (AF), alcohol and naltrindole treated (AF+NTD), and alcohol and naltrexone treated (AF+NTX) neonatal male and female rat pups at PND 6 by flow cytometry. Bar graphs show mean or median fluorescence intensity of staining of IBA-1 (A,B), MOR (C,D), DOR (E,F), TLR4 (G,H), p-38 MAPK (I,J), p-JNK (K,L), p-AKT (M,N), and NF-ΚB (0,P) from microglia isolated from each treatment group from male and female neonates. Data are represented as Mean ± SEM (n= 3-7) and were compared by one-way analysis of variance (ANOVA) and Newman-Keuls posttest. Differences between grroups are shown by lines with *p* values on the top of bar graphs.


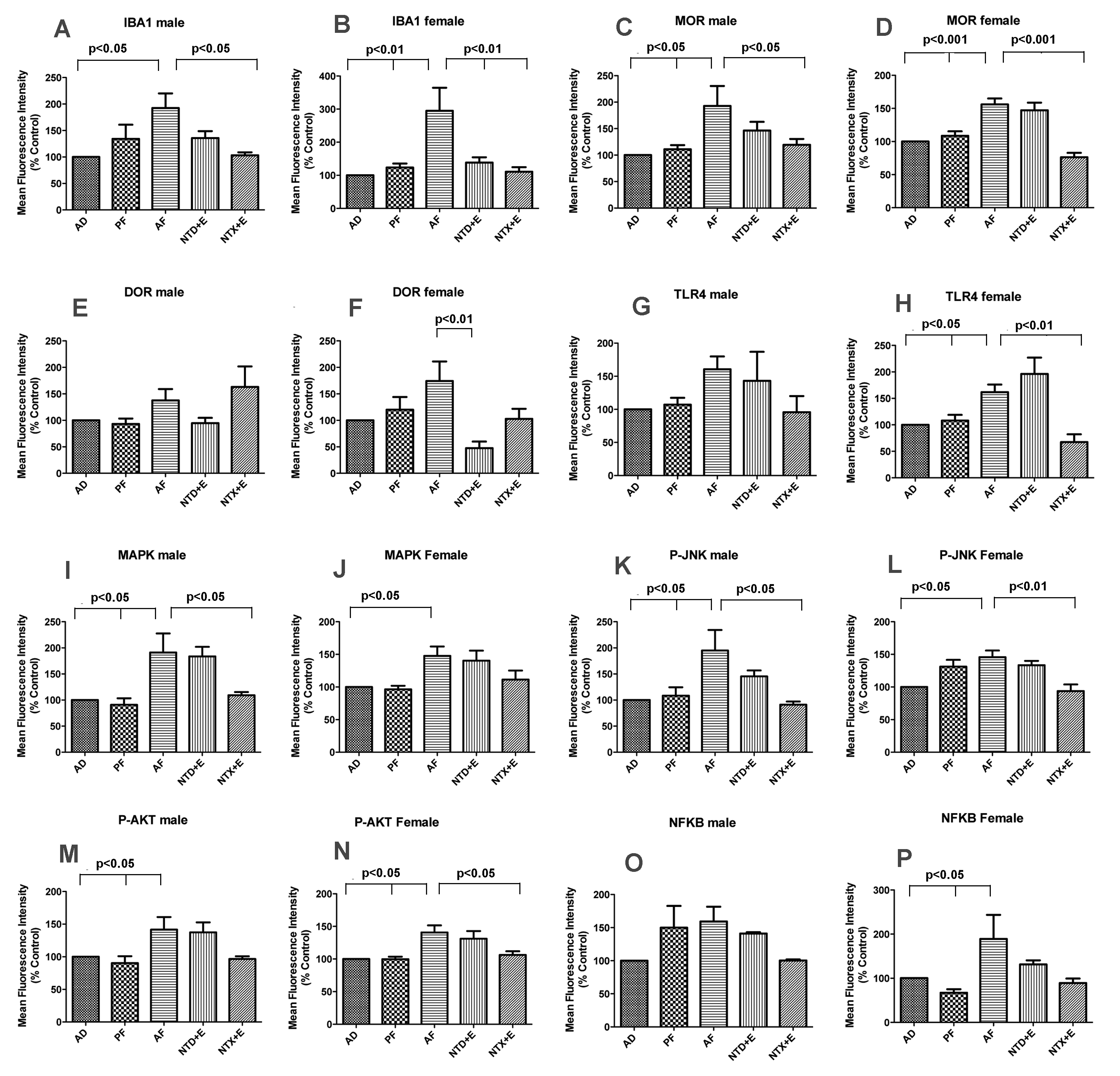

Supplement: Additional file 1: Figure S1. — Neonatal alcohol effects on microglial opioid receptors and TLR4 pathway proteins in the mediobasal hypothalamus of male and female neonates. Showing protein quantification in isolated microglia from ad libitum (AD), pair-fed (PF), alcohol-fed (AF), alcohol-fed and naltrindole-treated (AF + NTD), and alcohol-fed and naltrexone-treated (AF + NTX) neonatal male and female rat pups at PND 6 by flow cytometry. Bar graphs show mean or median fluorescence intensity of staining of IBA-1 (A, B), MOR (C, D), DOR (E, F), TLR4 (G, H), p-38 MAPK (I, J), p-JNK (K, L), p-AKT (M, N), and NF-κB (O, P) from microglia isolated from each treatment group from male and female neonates. Data are represented as mean ± SEM (n = 3–7) and were compared by one-way analysis of variance (ANOVA) and the Newman-Keuls posttest. Differences between groups are shown by lines with p values on the top of bar graphs. (DOCX 964 kb) [file 12974_2017_844_MOESM1_ESM.docx]
